# Optimal Operation of an Industrial Dividing Wall Column through Multiparametric Programming

**DOI:** 10.1021/acs.iecr.3c00836

**Published:** 2023-09-07

**Authors:** Iosif Pappas, Rahul Bindlish, Moustafa Ali, Efstratios N. Pistikopoulos

**Affiliations:** † Artie McFerrin Department of Chemical Engineering, Texas A&M University, College Station, Texas 77843, United States; ‡ Texas A&M Energy Institute, Texas A&M University, College Station, Texas 77843, United States; § Technical Expertise and Support Technology Center, The Dow Chemical Company, Houston, Texas 77077, United States

## Abstract

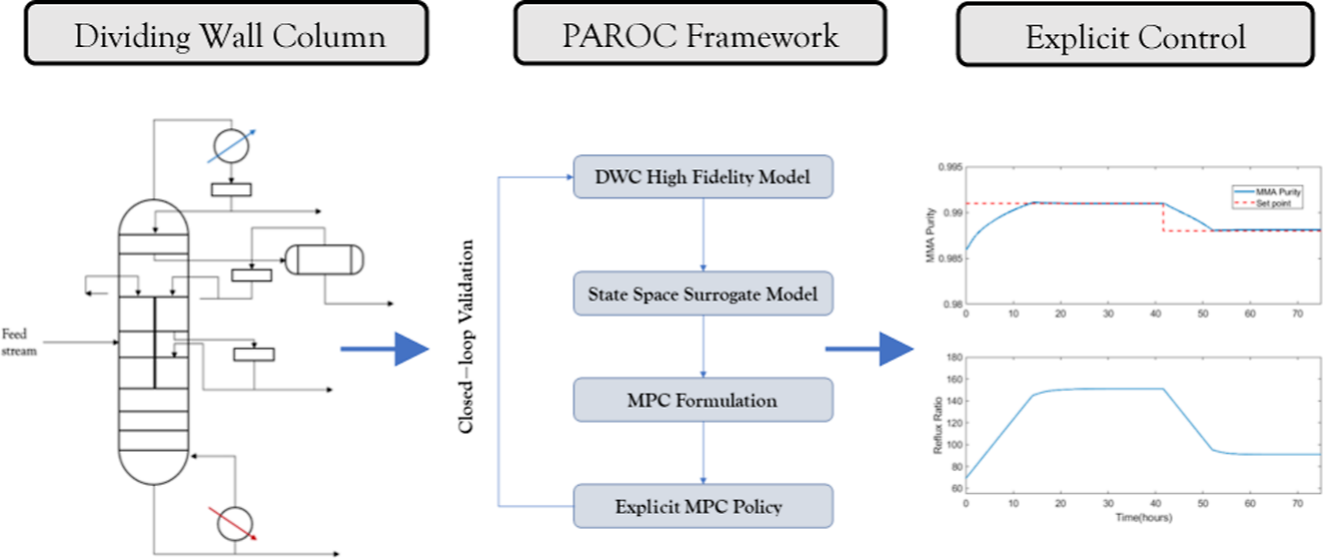

In this contribution, we present a high-fidelity dynamic model of an industrial dividing wall column and the application of explicit model predictive control for its regulation. Our study involves the separation of methyl methacrylate from a quaternary mixture. The process includes a dividing wall column coupled with a decanter, which results in highly concentrated methyl methacrylate and water streams from the middle side draw of the column and the decanter, respectively. An equation-oriented mathematical model of the process is developed and presented in detail, where non-ideal thermodynamic calculations are adopted to describe the complex nature of the component interactions. The operability of the process is enhanced by the synthesis and application of an explicit model predictive controller, which is used to track the purity specifications of the product. Our results demonstrate that the proposed modeling and control approach can be utilized for the optimal online operation of the studied system.

## 1 Introduction

Traditionally, the separation of multicomponent mixtures with distillation is performed by employing a sequence of distillation columns. Specifically, for a system with *n* components, *n* – 1 distillation columns are required at minimum for the separation of the mixture. Dividing wall columns (DWC) are advantageous alternatives for multicomponent separations compared to classical distillation trains. DWCs achieve efficient multicomponent separations by having a vertical wall in the column. The key advantage of DWCs compared to classical distillation is the absence of remixing: with sequential distillation, significant remixing of the components occurs, and hence, the energy requirements for the separation are dramatically enhanced. This effect is not as prevalent in DWCs due to the presence of the dividing wall. Apart from that, the absence of reboilers, condensers, and column sections in DWCs results in a substantial reduction in capital costs and space reduction. Overall, DWCs offer the potential of 30% reduction in investment and operating costs, and for this reason, major industries have adopted them in their operations.^1^

Apart from the numerous studies published in academic publications, the benefits of DWCs have allowed for their commercialization in different settings that have also been reported in the open literature. Even though DWC applications were not commercially adopted until 1985, they are nowadays considered to be a mature technology. Based on the contribution by Parkinson,^2^ tens of DWCs are built each year, while more than 100 are in operation worldwide. Namely, BASF AG operates more than 50 divided wall columns around the world, while ExxonMobil’s Fawley refinery operates a xylene recovery DWC, achieving more than a 50% energy savings and improved product purity. Furthermore, DWC technology has been integrated in several process schemes, with Sasol incorporating it into its Fischer–Tropsch process, UOP LLC offering it in its PACOL process for the dehydrogenation of long-chain paraffins, and Uhde GmbH commercializing DWCs as extractive distillation processes for the production of benzene, toluene, and xylene. Dow has operated a DWC pilot plant in Midland,^3^ while it has patented technology, such as for purifying methyl methacrylate with a reported purity of 98%. The mixture consisted of methyl methacrylate, methanol, water, and oligomers of methyl methacrylate.^4^

Despite the above and the wide acceptance of the process in industry, the ability to effectively regulate the operation of DWCs poses a significant hurdle in their further implementation.^5^ This is mainly due to the intensified nature of the process that leads to the loss of degrees of freedom to control the system compared to a series of columns. In addition to that, challenges are related to the significant interactions between the components by operating multiple operations in a single physical system. Consequently, a reason that makes the adoption of DWC technology in the industry slow is operability challenges, which result in flexibility issues.^6^ Hence, controlling such intensified processes is not a straightforward task.

Several publications have also appeared in the open literature with a focus on the regulation of DWCs, starting from regulatory control schemes to advanced control strategies. DWC designs require stable operation while maintaining product quality.^5^ Starting from the former, Yu et al.^7^ proposed an azeotropic DWC design for the dehydration of *tert*-butanol using cyclohexane as an entrainer. The Tray temperature control strategy was proposed using proportional-integral (PI) controllers. This selection was based on a sensitivity analysis on the effectiveness of manipulating the liquid split ratio to maintain the product quality. The control structure proposed was able to account for disturbances, maintaining the targeted product quality. Moreover, Sun et al.^8^ presented three control strategies for an extractive DWC (EDWC) design for the separation of benzene and cyclohexane processes. The control strategy involving PI temperature controllers without using the vapor split ratio as a degree of freedom was recommended. It showed the effectiveness of handling the disturbances while maintaining the product’s quality. In addition to that, Feng et al.^9^ proposed a PI control and a model predictive control (MPC) approach for separating 2-methoxyethanol and toluene using dimethyl sulfoxide as the entrainer in an EDWC based on temperature difference. The control strategy used temperature control to regulate the system. The dynamic response of the MPC scheme showed a much better performance, handling up to 20% disturbance in the feed flowrate compared to the PI scheme.

Van Diggelen et al.^10^ proposed a multi-loop control strategy for an industrial DWC design separating the ternary mixture of benzene-toluene-xylene. The authors performed a comparison of various control strategies. Four PID control strategies were proposed, and comparisons with multiple input, multiple output (MIMO) model-based controllers were made. The results showed that the MIMO controllers were superior in regulating the studied system. The effectiveness of model-based control was also exhibited by the work of Dohare et al.^11^ The authors modeled a DWC design for the separation of the same mixture system. The nonlinear dynamic model was controlled using a MPC configuration to control the three product compositions by indirectly controlling the temperature of the uppermost tray, bottom stage, and side stream withdrawal tray. This was achieved by manipulating the reflux ratio, side-stream flowrate, and reboiler heat duty. With a 10% disturbance in the feed, the MPC configuration showed a good performance with a settling time of 1.5 h compared to 3–4 h in the PI controller. Furthermore, a composition control scheme utilizing MPC strategies for an azeotropic DWC separating furfural–water mixture was proposed by Qian et al.^12^ The MPC scheme was able to achieve a low settling time of 2.20 h compared to 4.4 h in the case of the PI loop given a 20% disturbance in the feed flow.

Leal et al.^6^ utilized the Plantegrity NMPC system^13^ for the design of a control strategy for the DWC separating benzene–toluene–xylene mixture.^14^ The model-based approach was able to regulate the system using four manipulated variables (reflux ratio, liquid split, heat duty, and side stream molar flow rate). Rodríguez et al.^15^ established a decentralized control strategy as well as an MPC for the DWC design studied by Kiss and Ignat^16^ for concentrating and dehydrating ethanol using ethylene glycol as a mass separating agent. Level, pressure, and composition were selected as control variables. MPC showed sufficient performance with a smooth response on ethylene glycol composition. Finally, excellent overviews on the topic of control for DWCs can also be found in the research works of Donahue et al.^5^ and Kiss and Bildea.^17^

In recent years, there has been an evolution of smart manufacturing initiatives that has been taking place. This is highlighted in not only in academic but also in industrial applications, where a key focus is on computational performance, digitization, and availability and access to data quickly on demand. A fundamental element in such a framework is the seamless connectivity and rapid decision-making between the components of each process, where frequently optimal solutions need to be calculated on a cloud computing service. As described in the previous paragraph of this literature review, MPC has been shown to be a suitable control strategy to regulate DWCs. In a typical MPC application, the goal is to find the optimal sequence of control actions that optimize a performance criterion over a finite prediction horizon. This requires solving an optimization problem online, where future control actions are determined subject to the dynamics of the system and constraints. Because only the first control action is applied to the system in a closed-loop, this process needs to be repeated at every time step. Consequently, significant computational power is required. Instead of solving the aforementioned problems online, the problem can be treated and exactly solved as a multiparametric programming problem.^18^ Multiparametric programming can provide the optimal solution of an optimization problem as an analytic function of its uncertain parameters, which appear in the optimization formulation. In an MPC setting, the control actions (decision variables) are expressed as a function of the initial states of the system, which are treated as a part of the uncertain parameters. Such a technique is termed multiparametric/explicit MPC (mpMPC). mpMPC has already been proven to be a valuable tool for the regulation of intensified systems (Tian et al.^19^) by applying the PARametric optimization and control (PAROC) framework given by Pistikopoulos et al.^20^ However, the benefits of adopting an mpMPC strategy are not limited to the rapid computation of the control actions, whose absence may be connected to a significant financial cost if optimization problems need to constantly be solved on a cloud computing service. By having the full explicit solution of the MPC before even the closed-loop simulation has occurred, it can support extensive analysis of the behavior of the system and the identification of operating regions where unsatisfactory performance is observed. Apart from that, the derivation of explicit control laws can be used in various other studies, such as the integration of design, control, and scheduling, robust control, and multilevel optimization. The interested reader is referred to a review that discusses the benefits of mpMPC and multiparametric optimization in general.^21^ To our knowledge, mpMPC has not been applied to DWCs for MMA purification or to DWCs in general. In this work, we examine the application of PAROC to an industrial DWC system. With this case study, we attempted to consider a practically relevant problem, whose modeling includes significant complexity. However, it has not been an objective of ours to compare the performance of the MPC with a PI/PID control system, as we wanted to focus on the performance of MPC and also because such comparisons have already been made in published literature.

The remainder of the paper is structured as follows: in the next section, the problem statement of the study is presented, while in Section 3, the steps used for the application of the PAROC framework are exhibited. Section 4 includes the results of our study, while in Section 5, we conclude.

## 2 Problem Statement

### 2.1 Process Description

**Figure 1:**
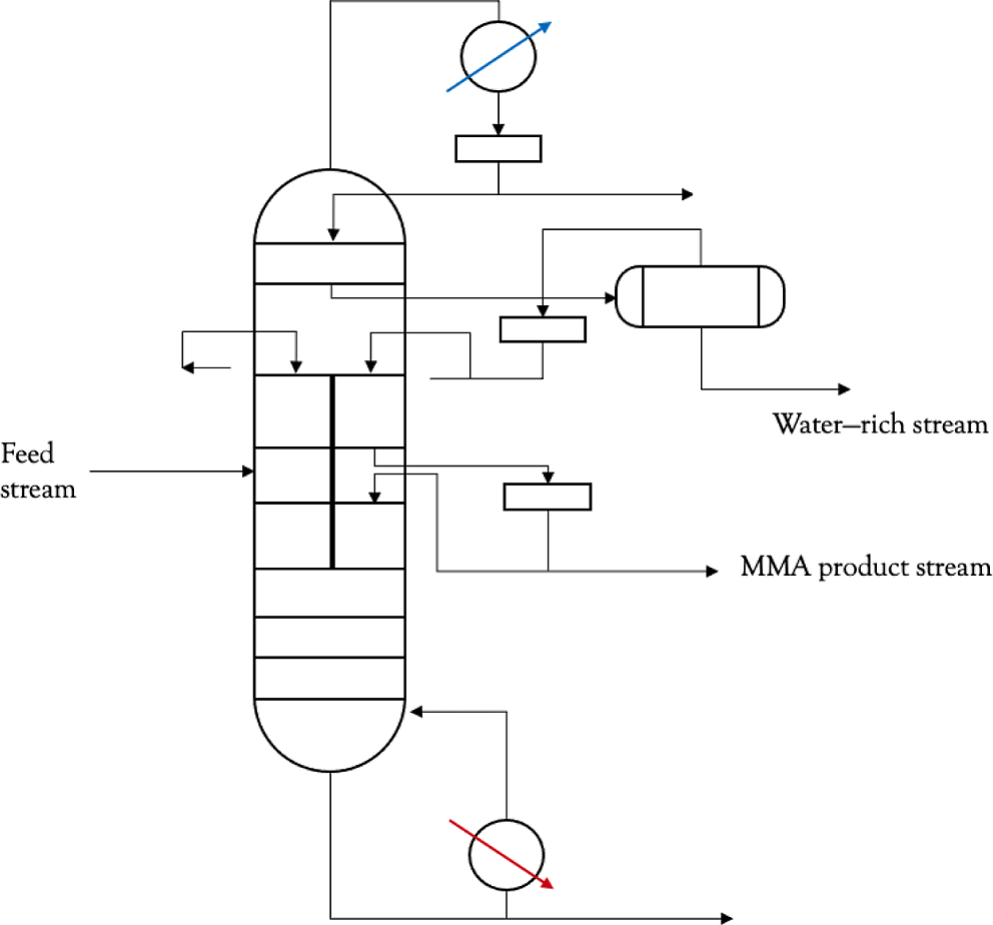
Schematic of the patented DWC process: adapted from Jewell et al.^4^

The present study is concerned with the dynamic modeling and regulation of a DWC for the purification of MMA. The description of the process is defined based on the patent by Dow Global Technologies, as detailed in Jewell et al.^4^ The invention focuses on the separation of a quaternary mixture consisting of MMA, water, methanol, and MMA oligomers. The oligomers of MMA include the dimer of MMA and smaller amounts of higher oligomers. The feed stream enters the DWC, where the highest concentration of MMA is achieved and is withdrawn at the middle draw of the column. Apart from the DWC, the overall process additionally includes a decanter, which facilitates the separation of two liquid phases: an MMA-rich oil phase and a water phase. The aforementioned liquid–liquid separation results in a highly concentrated water stream, while the dewatered stream is recycled back to the DWC. A schematic representing the process is shown in Figure 1.

The given process design has been shown to have a 7.6% increased recovery of the desired MMA product compared to the conventional two column design distillation process while utilizing the same number of trays and energy requirements.

### 2.2 Research Objectives

Given the advantageous performance of the present DWC design, it is necessary to demonstrate that it can be operated in real time. Steady-state simulations of the process have successfully been presented.^4,22^ However, there exist several modeling and operability challenges associated with DWCs and intensified designs in general due to the complex system interactions and the reduction of available manipulated variables.^23^ Hence, the objectives of this work are the following:

- Develop a high-fidelity dynamic model to describe the purification of MMA from a mixture of MMA, water, methanol, and MMA oligomers.
- Synthesize an mpMPC that regulates the system at the desired % MMA purity in the middle product stream. The setpoint for the purity of the product is set to 99.1%.

The validation that the process can be dynamically operated will provide further confidence in adopting this technology in practice.

## 3 Methodology

**Figure 2:**
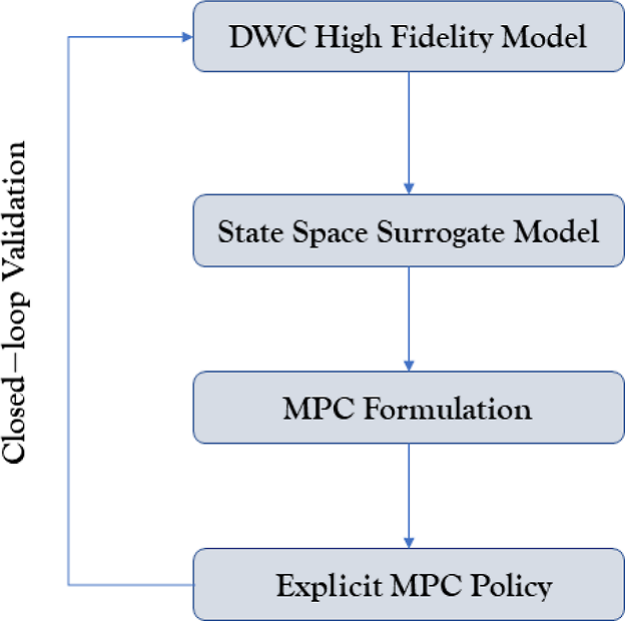
Schematic of the PAROC framework as applied to the present DWC study.

The methodology that we will follow for the derivation of the optimal operational policy is the one described by the PAROC framework.^20^ PAROC consists of four steps: (i) high-fidelity modeling, (ii) model approximation, (iii) the formulation of the MPC problem, and (iv) its explicit solution through multiparametric programming. This framework has already been successfully applied to numerous systems, including intesified processes.^19^ Except for advanced control, PAROC offers a roadmap for various other operational studies using multiparametric programming, such as scheduling and state estimation.^21^ A schematic of the PAROC framework is shown in Figure 2.

Given the complex nature of the DWC, our first goal is to derive an accurate representation of the system. Hence, we try to include in our high-fidelity model as much detail as possible. Nevertheless, given that mpMPC is a model-based formulation, directly incorporating the large-scale dynamic model in the mpMPC formulation is computationally challenging. For this reason, in the second step of PAROC, we build a surrogate model that balances the computational complexity and accuracy of the model so that the solution of the explicit MPC problem is viable. After formulating the MPC problem, it is subsequently solved, and the optimal control policy is derived analytically. Finally, because our control solution is based on the surrogate model, we validate it by applying it back to the original high-fidelity model. As a result, the online operation of the system in a closed loop is facilitated.

### 3.1 High-Fidelity Modeling

**Figure 3:**
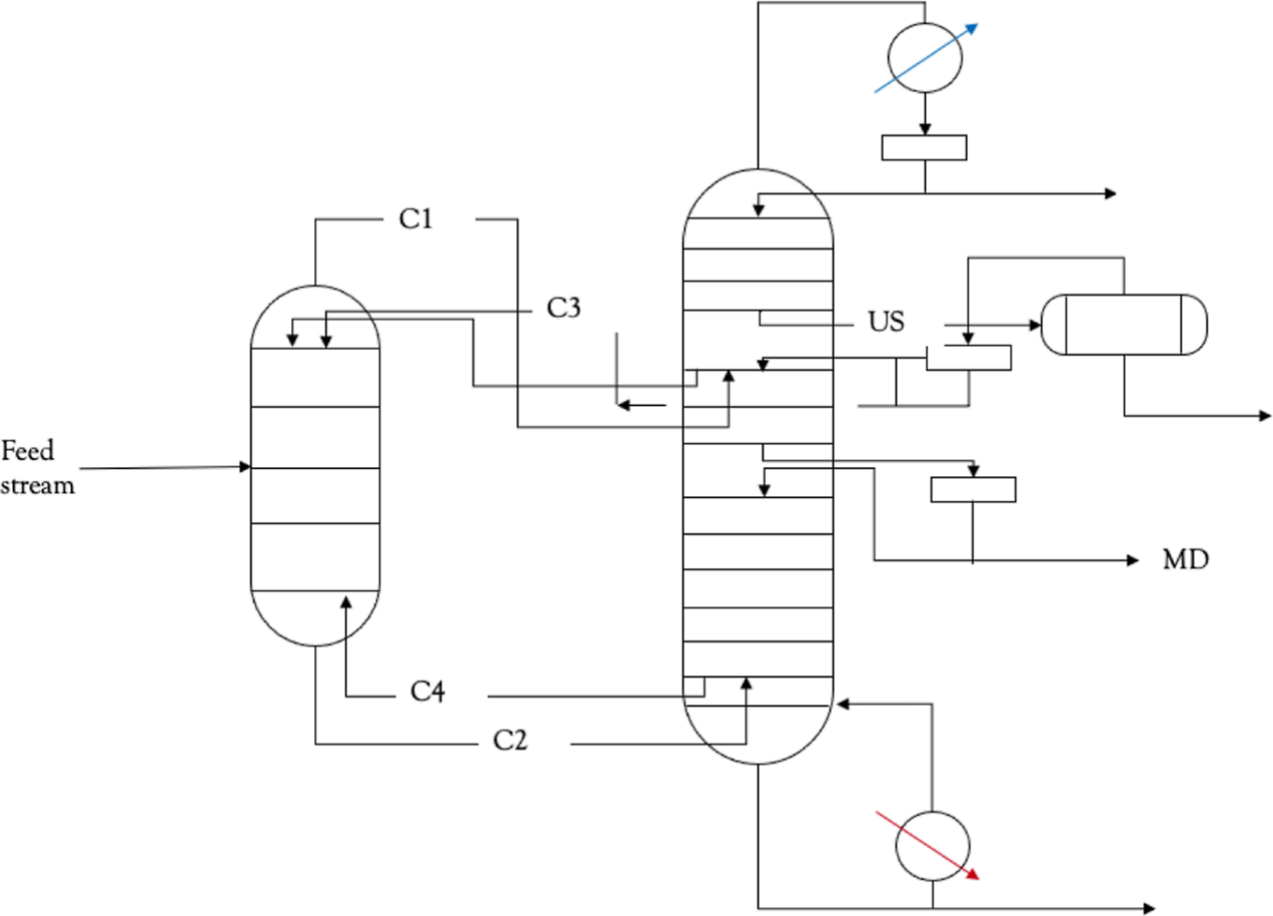
Schematic of the Petlyuk process based on which the high-fidelity model was developed.

The assumptions for the development of the equation-oriented high-fidelity model that include the process design and other parameters are based on the patent and the corresponding steady-state model that have recently been published.^4,22^ The DWC is modeled as the thermodynamically equivalent Petlyuk column, which consists of a prefractionator and is integrated with the main column of the system, followed by the decanter. A representation of the process is exhibited in Figure 3, while its parameters are exhibited in Table 2.

**Table 1:** Variable Description and Value for Open-Loop Simulation of the DWC System

Unit	Variable	Value
Prefractionator	Number of trays	5
	Feed tray	3
	Feed flowrate (kmol/h)	200.96
	Feed temperature (K)	298.15
	Feed pressure (atm)	1
	Pressure (atm)	0.06
Main Column	Number of trays	15
	Pressure (atm)	0.06
	Middle draw product tray location	8
	Upper side draw tray location	4
	Upper side flowrate (kmol/h)	376
Condenser	Pressure (atm)	0.06
	Reflux ratio	69
Reboiler	Pressure (atm)	0.06
	Bottom flowrate (kmol/h)	9.9
Decanter	Pressure (atm)	0.06
	Temperature (K)	323.15
	Decanter water stream (kmol/h)	9.1
	Decanter oil stream (kmol/h)	366.9

**Table 2:** Stream Information and Connections of the DWC System

Stream	Phase	Source^a^	Destination	Flowrate (kmol/h)
C1	Vapor	Prefractionator 1	Main Column 5	54.9
C2	Liquid	Prefractionator 5	Main Column 14	415.4
C3	Liquid	Main Column 5	Prefractionator 1	43.6
C4	Vapor	Main Column 14	Prefractionator 5	42.5
US	Liquid	Main Column 4	Decanter	376.0
MD	Liquid	Main Column 8	Product	176.0

a The number next to the prefractionator or the main column indicates the tray number where the stream is sent/withdrawn.

Additionally, we provide the stream connections for the system:

The main features of the model are:

- Dynamic material and energy balances for each tray of the system.
- Calculation of material and energy holdups.
- Consideration of non-ideal liquid–liquid and vapor–liquid equilibrium for the processing units of the system. The UNIFAC model is utilized for the calculation of the activity coefficients, vapor pressures, enthalpies, and densities of the components of the system.
- Capability of adjusting the efficiency (ideality) of the tray separation by manipulating the Murphree tray efficiency.
- Description of the liquid level and the hydraulics on each tray.

The selected modeling platform for the process is the equation-oriented gPROMS Modebuilder.^24^ For the calculation of the various thermodynamic properties, gPROMS is coupled with KBC’s Multiflash 6.1. The overall model includes 1232 equations with design and operating parameters in Table 1. The model is described in detail in the Supporting Information of this manuscript. An element that has been crucial in this work was able to accurately describe the fact that the in the middle of the column, the highest molar fraction of MMA exists. In this respect, the UNIFAC thermodynamic package has been used that presented a satisfactory agreement with the results of the steady-state simulation. The development of this model is the foundation of this study since it allows for extended open-loop simulations and sensitivity analysis to evaluate its performance. It has to be noted that it is not possible for our model to be identical to the already developed steady-state models, as they have been developed in different platforms.

### 3.2 Surrogate Modeling

Given that the direct incorporation of the dynamic model in an optimization formulation is challenging, in this step we build a surrogate model for the DWC which would be able to balance accuracy and computational complexity.^25^ In this step, we also want to make a suitable pairing for our controlled variable (MMA purity) and the manipulated variable. We performed sensitivity analyses of various variables that can influence the system, and the reflux ratio of the main column has the biggest effect on the MMA purity. Based on that, we generated input–output data of our system by varying the reflux ratio and observing the corresponding values for the MMA purity.

**Figure 4:**
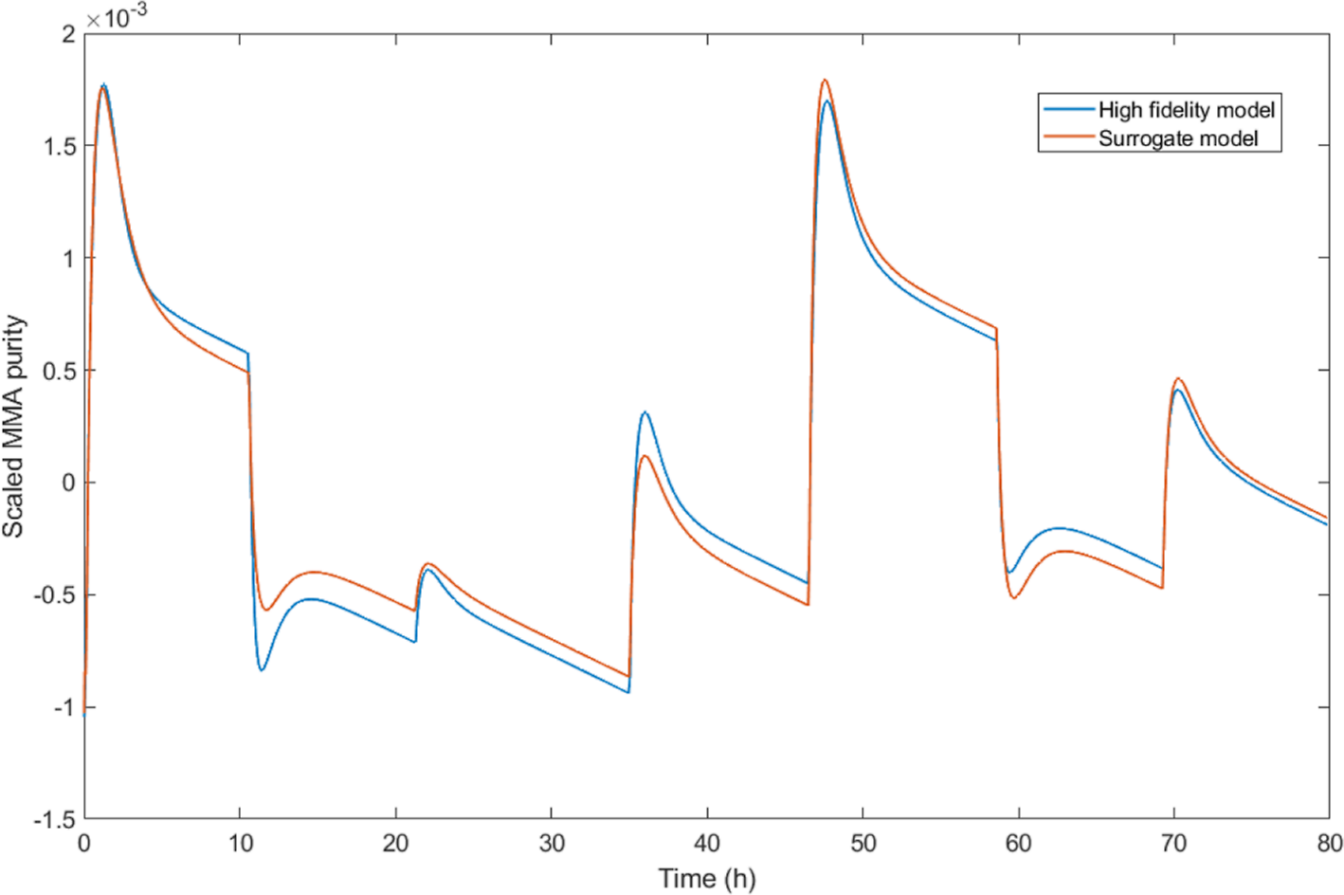
Comparison of the high-fidelity model and the surrogate model for the DWC process.

The discrete time invariant linear state space surrogate model, which was identified is of the following form
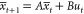
1a
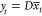
1bwhere the matrices *A*, *B*, *D* are defined in the Supporting Information of the article. The model consists of five pseudostates, *x̅*, one input (reflux ratio), *u*, and one output (MMA purity), *y*, while the timestep for the development of the model is 100 s. As can be observed from Figure 4, the derived approximate model is able to describe the variations of MMA purity based on the reflux ratio changes. By selecting the reflux ratio as the manipulated variable to regulate the product purity, a single-input, single-output system setup is implied. Even though one of the main advantages of explicit MPC is the consideration of multiple-input, multiple-output (MIMO) systems, the inclusion of more variables in the control design would unnecessarily increase the computational complexity. Hence, this model and a single input will be used for the construction of the mpMPC in the next step of the framework.

### 3.3 Explicit MPC

The goal of the mpMPC formulation is to minimize the deviation of the MMA purity from the desired setpoint. This is expressed through the following optimization formulation
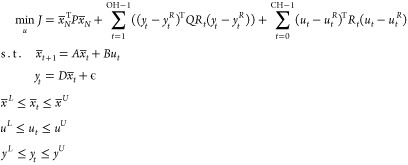
2where OH and CH are the output and control horizons, respectively, *QR*_*t*_ and *R*_*t*_ are the weights penalizing the deviation of the outputs and inputs from their reference trajectories, *y*_*t*_^*R*^ and *u*_*t*_^*R*^, while *P* is a terminal weight matrix derived from the solution of the Riccati equation. The pseudostates, the inputs, and the outputs have lower and upper bounds denoted with the superscripts *L* and *U,* respectively. The problem above must be repetitively solved at each time of the horizon, where at each sampling instance, the vector of control inputs for all future steps is calculated, but only the first control input is applied. As soon as this is achieved, the horizon is shifted by one step and the problem is resolved. Consequently, feedback is implicitly facilitated. We note that we have assumed that there is no base control layer and that only the MPC application is used for the regulation of the system. The reason for that was that we wanted to focus on the demonstration of the applicability of the MPC as well as the evaluation of its performance. However, frequently in process control applications, an MPC is used as a supervisory control layer together with regulatory PI/PID controllers.

Given that we have a linear model in the MPC formulation, we can express the future pseudostates of the system as a function of their initial values as well as the control actions through the following expression
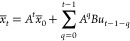
3

As soon as this is incorporated in expression (2), the MPC problem is equivalent to the multiparametric quadratic programming problem, where the vector of the control inputs, *u*, is again the vector of the decision variables, while  is the vector of the uncertain parameters of the problem.
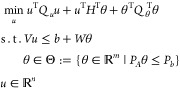
4

By reformulating the problem from the formulation (1b–3) the problem is solved once and offline through multiparametric programming techniques, and hence, the online computational cost is minimized.^26^ The optimal explicit solution is of the following form
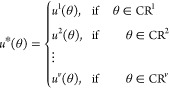
5

Depending on where the current value of the uncertain parameters lies (denoted with CR), the corresponding value of the optimal solution is selected.

In our formulation, we enforce the bounds on the pseudostates, inputs, and outputs as follows:
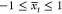
6a
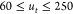
6b
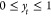
6c

The control horizon is set to 3, while the output horizon is 5. This was achieved through an iterative process by attempting to strike a balance between the predictive ability of the control and computational performance. Smaller values for the control and output horizons resulted in challenges in regulating the column.

### 3.4 Closed-Loop Validation

**Figure 5:**
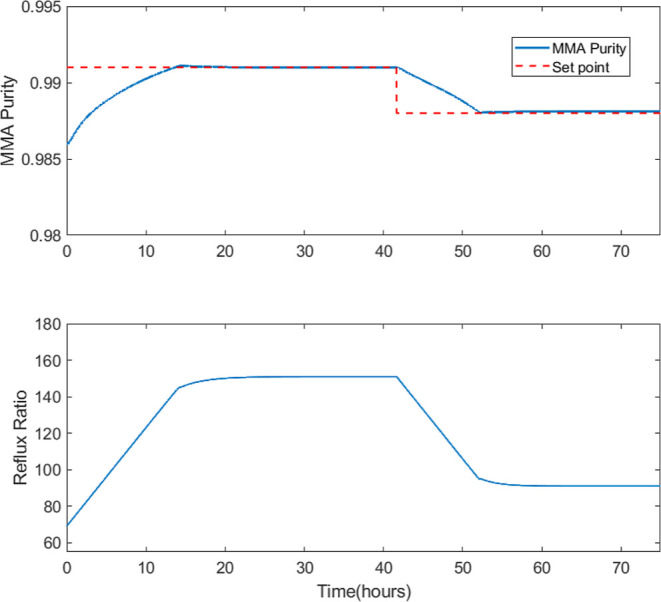
Closed-loop simulation of the DWC process for two different setpoints. Top figure: MMA purity response. Lower figure: reflux ratio values.

The control objective of this study is to demonstrate that the controller can be regulated at the desired setpoint. Note that this is a crucial step since the controller design has been based on a surrogate linear model instead of the high-fidelity model, and as a result, model deviations can lead to unacceptable operation.^27^ The open-loop steady-state simulation for a value of the reflux ratio of 69 provides an output for the MMA purity to be 98.6%. Given this initial point, the system is to drive to the setpoint of 99.1% and subsequently move it back to 98.8%. The results of the closed-loop simulation are shown in Figure 5.

As it can be observed, the controller manages to control the process around the setpoints. Hence, our model has the flexibility of separating MMA depending on the product specification. For the controller to achieve that, the reflux ratio needs to be significantly increased so that the majority of the vapor product leaving the top of the column is returned. That is to be expected since the nominal design already requires a large value for the reflux ratio (69). Another key point to be highlighted is that the process has relatively slow dynamics since it takes multiple hours to reach each of the desired operating targets.

## 4 Conclusions

In this study, we presented the regulation of a DWC that separates MMA from a multicomponent mixture of MMA, methanol, water, and MMA oligomers, utilizing the PAROC framework. This industrial process was based on a patented technology which had been demonstrated to have superior performance compared to the conventional sequential two-distillation column process. A high-fidelity model of the process was developed which accurately described the open-loop operation of the system. The resulting model consisted of a large-scale system of differential and algebraic equations. Based on that, a suitable data-driven approximate model was developed, which allowed for its use in an explicit MPC formulation. The derived explicit control policy was applied back to the high-fidelity system, which showed that the system can be regulated at the desired MMA purity levels by manipulating the reflux ratio of the column. Hence, these results provide confidence in adopting this technology in practice. That is particularly crucial, given that intensified processes are linked with substantial challenges regarding their real time operation.

## Supporting Information Available

Here, we present the detailed dynamic model for the operation of the DWC along with the surrogate model matrix; high-fidelity model notation are presented with a TOC graphic for the full paper; and presented notations refers solely to the high-fidelity DWC model. This information is available free of charge via the internet at this link (PDF)
