# Supplementary material for: Optimal Operation of an Industrial Dividing Wall Column through Multiparametric Programming
Source: Ind Eng Chem Res. 2023 Sep 7;62(37):15029–35. doi: 10.1021/acs.iecr.3c00836 (PMC10863063; doi:10.1021/acs.iecr.3c00836)
Supplement: Supplementary file 1 — ie3c00836_si_001.pdf [file ie3c00836_si_001.pdf]

Optimal Operation of an Industrial Dividing Wall Column through Multiparametric Programming

Iosif Pappas,[†,‡] Rahul Bindlish,[¶] Moustafa Ali,[‡] and Efstratios N. Pistikopoulos[*,†,‡]

†*Artie McFerrin Department of Chemical Engineering, Texas A&M University, College Station, TX 77843, U.S.A.*

‡*Texas A&M Energy Institute, Texas A&M University, College Station, TX 77843, U.S.A.*

¶*Technical Expertise and Support Technology Center, The Dow Chemical Company, Houston, TX, 77077, U.S.A*

E-mail: stratos@tamu.edu

Phone: +1-979-458-0259

Supporting Information

High fidelity Model

Here, we present the detailed dynamic model for the operation of the DWC. The focus has been on developing a rigorous representation of the system with the goal to describe the complex relationships of the system.[?,?,?]

We start with the modeling of the prefractionator and the main column, which are modeled as a distillation column sections, fully connected between them. We denote each component with the index $i \in I$ and the number of trays to be $k \in K$.

The component molar balance is given by the following equation:

$$\frac{dM_{i,k}}{dt} = \sum_{feed} F_{feed} z_{i,feed} + L_{k-1}x_{i,k-1} + V_{k+1}y_{i,k+1} - L_k x_{i,k} + \\ -V_k y_{i,k} + R x_{i,d} \;\; i = 1, ...NC, \;\; k = 1, ..., N \tag{1}$$

where $M_{i,k}$ is the holdup at the kth tray of the ith component, and $L_{i,k}$ and $V_{i,k}$ are the corresponding liquid and vapor molar flowrates. The liquid and vapor molar fractions for each component and tray are given by $x_{i,k}$ and $y_{i,k}$ respectively, while $z_{i,feed}$ is the feed molar fraction at the feed stream. R denotes the reflux ratio and $x_{i,d}$ is the molar fraction of the distillate.

The holdup at each tray is described by the contributions of the holdups in the liquid and vapor phase M_k^l and M_k^v:

$$M_{i,k} = M_k^l x_{i,k} + M_k^v y_{i,k} \qquad i = 1, ...NC, \;\; k = 1, ..., N \tag{2}$$

Similarly we can write the energy balances for each tray:

$$\frac{dU_k}{dt} = \sum_{feed} F_{feed,k} h_{feed} + L_{k-1}h_{k-1}^l + V_{k+1}h_{k+1}^v \\ -L_k h_k^l + V_k h_k^v + R_k h_{l,d} \qquad k = 1, ..., N \tag{3}$$

where U_k is the molar internal energy holdup, and h_k^l and h_k^v are the liquid and vapor enthalpies. The energy coming back from the reflux drum to the column is given by $h_{l,d}$. We also write the total energy balance which is a linear combination of the enthalpy contributions the liquid and vapor phases.

$$U_k = M_k^l h_k^l + M_k^v h_k^v - 0.1 P_k Vol_{tray} \qquad k = 1, ..., N \tag{4}$$

P_k is the pressure at each tray of the column. In this work, we assume the pressure profile

in the column is constant:

$$P_k = P \qquad k = 1, ..., N \tag{5}$$

Additionally, the volume of each tray is denoted with V_{tray} and is connected with the molar density of the liquid and the vapor phase, ρ_k^l and ρ_k^v, as follows:

$$\frac{M_k^l}{\rho_k^l} + \frac{M_k^v}{\rho_k^v} = Vol_{tray} \qquad k = 1, ..., N \tag{6}$$

The liquid level on each tray can be calculated by the following set of equations:

$$Level_k = \frac{M_k^l}{\rho_k^l A_{tray}} \qquad k = 1, ..., N \tag{7}$$

A_{tray} is the surface area of each tray, through which the free volume between the trays can be found:

$$Vol_{tray} = A_{tray} S \tag{8}$$

S denotes the spacing between the trays.

As mentioned for the vapor-liquid equilibrium calculations the UNIFAC model will be used to calculate the fugacity and activity coefficients of each component of the quarternary mixture. Specifically, assuming that the vapor and the liquid phase are in equilibirum at each tray, we have:

$$\Phi_{i,k}^v y_{i,k}^* = \Phi_{i,k}^l x_{i,k} \qquad i = 1, ...NC, \; k = 1, ..., N \tag{9}$$

In the above relationship $\Phi_{i,k}^l$ and $\Phi_{i,k}^v$ are the liquid and vapor activity coefficients respectively at each tray.

We also allow that the tray efficiency might not exhibit ideal behavior and for this reason we introduce the Murphree efficiency relationship:

$$y_{i,k} = y_{i,k+1} + \epsilon_{i,k}(y^*_{i,k} - y_{i,k+1}) \qquad i = 1, ...NC,\ k = 1, ..., N \tag{10}$$

where $\epsilon_{i,k}$ stands for the Murphree tray efficiency. Nonetheless, in this work we assume that $\epsilon_{i,k} = 1$, $\forall i \in NC, k \in N$.

Furthermore, we impose that the summations of the molar fractions in the liquid and the vapor phase are equal to one:

$$\sum_{i=1}^{NC} x_{i,k} = 1 \qquad k = 1, ..., N \tag{11}$$

$$\sum_{i=1}^{NC} y_{i,k} = 1 \qquad k = 1, ..., N \tag{12}$$

The liquid outlet flowrate are connected with the liquid level in each tray, as described by the following relationship:

$$L_k = \begin{cases} 0, \text{ if } Level_k \leq Height_{weir} \\ 1.84 \cdot \rho^l_k \cdot Length_{weir} \cdot (Level_k - Height_{weir})^{1.5}, \text{ otherwise} \end{cases} \tag{13}$$

and the geometry of the columns are described by including the distance between each tray, *Space*, as well as the diameter of the column, D_{col}. The area of the column is given by A_{col}.

$$Vol_{tray} = Space A_{tray} \tag{14}$$

$$A_{col} = \frac{\pi}{4} D^2_{col} \tag{15}$$

Accurate calcuations of the thermodynamic properties of the components in the column are crucial for the description of its operation as well as for its simulation. The software Multiflash 6.1. by KBC is used to calculate the following properties:

$$h^l = h^l(P, T, \mathbf{x}) \tag{16}$$

$$h^v = h^v(P, T, \mathbf{y}) \tag{17}$$

$$\rho^l = \rho^l(P, T, \mathbf{x}) \tag{18}$$

$$\rho^v = \rho^v(P, T, \mathbf{y}) \tag{19}$$

$$\tilde{\rho}^l = \tilde{\rho}^l(P, T, \mathbf{x}) \tag{20}$$

$$\tilde{\rho}^v = \tilde{\rho}^v(P, T, \mathbf{y}) \tag{21}$$

$$\Phi_i^l = \Phi^l(P, T, \mathbf{x}) \qquad i = 1, ..., NC \tag{22}$$

$$\Phi_i^v = \Phi^v(P, T, \mathbf{y}) \qquad i = 1, ..., NC \tag{23}$$

In the equations above $\tilde{\rho}^l$ and $\tilde{\rho}^v$ are used to denote the mass densities of the liquid and vapor.

Similary to column sections, the reboiler, condenser as well as the decanter are modeled column sections with one tray. However, for the decanter we assume that there is not any holdup and for the liquid-liquid equilbrium between the oil and water phase the following relationship holds:

$$\Phi_{i,k}^v x_{i,decanter} = \Phi_{i,k}^l x_{i,decanter} \qquad i = 1, ...NC, \ k = 1, ..., N \tag{24}$$

Surrogate Model

$$A = \begin{bmatrix} 0.9605 & 0.1873 & -0.0352 & -0.0014 & 0.0125 \\ -0.2512 & 0.3294 & -0.3897 & -0.0007 & 0.0891 \\ -0.0448 & 0.6425 & -0.4490 & -0.0701 & -0.2572 \\ 0.0127 & -0.0565 & -0.1347 & 0.9814 & -0.0142 \\ 0.0057 & -0.0332 & -0.0619 & -0.0463 & 0.8395 \end{bmatrix}$$

$$B = \begin{bmatrix} -0.0027 \\ -0.0145 \\ -0.0801 \\ -0.0069 \\ -0.0037 \end{bmatrix} \tag{25}$$

$$D = \begin{bmatrix} 0.0038 & 0.0017 & 0.0002 & -0.0097 & 0.0020 \end{bmatrix}$$

High Fidelity Model Nomenclature

The following notation solely refers to the high fidelity DWC model:

$\epsilon_{i,k}$ Murphree tray efficiency for component i on tray k

A_{col} cross-sectional area of the column

A_{tray} active tray area

D_{col} column diameter

F_{feed} feed flowrate

h^l_k molar liquid enthalpy at tray k

h^v_k molar vapor enthalpy at tray k

$Height_{weir}$ tray weir height

L_k liquid molar flowrate for tray k

$Length_{weir}$ tray weir length

$Level_k$ liquid level for tray k

$M_k^{l,v}$ molar liquid or vapor holdup for tray k

$M_{i,k}$ molar holdup of component i for tray k

P_k pressure for tray k

R reflux flowrate

$Space_{tray}$ Spacing between each tray of the column

T temperature

U_k molar internal energy holdup for tray k

V_k vapor molar flowrate for tray k

Vol_{tray} tray volume

$x_{i,k}$ liquid molar fraction of component i for tray k

$y_{i,k}$ vapor molar fraction of component i for tray k

$y^*_{i,k}$ equilibrium vapor molar fraction of component i

$z_{i,feed}$ molar fraction of component i in the feed

i index set for the components

k index set for the trays

Φ^l liquid fugacity coefficient

Φ^v vapor fugacity coefficient

ρ^l liquid molar density

ρ^v vapor molar density

$\tilde{\rho}^l$ liquid mass density

$\tilde{\rho}^v$ vapor mass density
